# Carbon footprint of the Brazilian diet

**DOI:** 10.11606/s1518-8787.2021055003614

**Published:** 2021-11-23

**Authors:** Josefa Maria Fellegger Garzillo, Priscila Pereira Machado, Fernanda Helena Marrocos Leite, Euridice Martinez Steele, Vanessa Fadanelli Schoenardie Poli, Maria Laura da Costa Louzada, Renata Bertazzi Levy, Carlos Augusto Monteiro

**Affiliations:** I Universidade de São Paulo Núcleo de Pesquisas Epidemiológicas em Nutrição e Saúde São Paulo SP Brasil Universidade de São Paulo. Núcleo de Pesquisas Epidemiológicas em Nutrição e Saúde. São Paulo, SP, Brasil; II Deakin University Institute for Physical Activity and Nutrition Melbourne Austrália Deakin University. Institute for Physical Activity and Nutrition. Melbourne, Austrália; III Universidade de São Paulo Faculdade de Medicina Departamento de Medicina Preventiva São Paulo SP Brasil Universidade de São Paulo. Faculdade de Medicina. Departamento de Medicina Preventiva. São Paulo, SP, Brasil

**Keywords:** Carbon Footprint, Basic Diet, Socioeconomic factors, Brazil

## Abstract

**OBJECTIVE:**

To estimate the carbon footprint of the Brazilian diet and of sociodemographic strata of this population.

**METHODS:**

Carbon footprint of the diet was estimated based on data from two 24-hour diet records, obtained in 2008 and 2009, from a probabilistic sample of the Brazilian population aged 10 years and over (n = 34,003) and on environmental impact coefficients of food and culinary preparations consumed in Brazil (gCO2e/kg). Means with 95% confidence intervals of food consumption (kcal/person/day) and the carbon footprint of the diet (gCO2e/person/day and in gCO2e/2,000kcal) were calculated for the population as a whole and for strata according to sex, age, income, education, macro-regions and Federative Unit. Linear regression models were used to identify significant differences (p < 0.05) in the dietary carbon footprint of different sociodemographic strata.

**RESULTS:**

The average carbon footprint of the Brazilian diet was 4,489gCO2e/person/day. It was higher for males, for the age group from 20 to 49 years and for the North and Midwest regions, and tended to increase with income and education. The pattern of association of footprint with sociodemographic variables did not change substantially with adjustment for differences in the amount of food consumed, except for a reduction in the relative excess of the footprint among males and an increase in the relative excess of the footprint in the Midwest region.

**CONCLUSION:**

The carbon footprint of the Brazilian diet exceeds by about 30% the footprint of the human diet, which could simultaneously meet the nutritional requirements of a healthy diet and the global goal of containing the increase in the planet's average temperature. The pattern of association of this footprint with sociodemographic variables can help identify priority targets for public actions aimed at reducing the environmental impacts of food consumption in Brazil.

## INTRODUCTION

The transition to a sustainable food system is a collective urgency, given the seriousness of global environmental changes and the impacts of food on ecological balance^1^. Brazil has assumed multilateral commitments such as eliminating hunger and combating climate change^2^, which requires the nutritional and environmental sciences to join forces to understand the multiple impacts of food^3^ and to build guidelines on adequate, healthy and sustainable diets under Brazilian conditions, clarifying the population and governments on how to protect nature^1^.

Studies on the impacts or environmental footprints of food require the availability of representative data on the population's food consumption and indicators that quantify the footprints of individual foods that make up the diet. These indicators, calculated using the product life cycle assessment methodology^4^, account for the use of natural resources and the load of pollutants released into the environment per kilogram of food, such as the coefficient of the food's carbon footprint, which quantifies the amount of atmospheric emissions of greenhouse gases. Based on studies of the environmental impact of food, dietary patterns such as the “Mediterranean diet” and the “vegetarian diet” are considered models for mitigating the negative effects of food on the environment^5-8^.

Environmental impact assessments of diets that cover the general population, as well as specific sociodemographic strata, are relevant because they identify both the critical points to change in diet practices as the population groups public policies should focus on for substantial reductions in environmental impacts.

In this article, based on data collected in a national survey on food consumption in Brazil, we estimate the carbon footprint of the diet consumed by the Brazilian population and the diet of sociodemographic strata of this population.

## METHODS

### Source of Food Consumption Data

The data on food consumption analyzed in this study come from the personal food consumption assessment module of the Family Budget Survey (POF) carried out by the Brazilian Institute of Geography and Statistics (IBGE) between May 2008 and May 2009 (POF 2008–2009)^9^.

The 2008–2009 POF used a complex sampling plan, by clusters, with geographic and socioeconomic stratification of all census sectors in the country, followed by random drawings of sectors in a first stage and households in a second. The number of sectors drawn in each stratum was proportional to the number of households in it. The selection of households in each sector was carried out by simple random sampling without replacement. The sample covered 55,970 households and the module for assessment of personal food consumption was applied to a random sub-sample of 13,569 households (24.3% of the total number of households studied)^9^.

The interviews conducted by POF 2008–2009 in each stratum of the sample were evenly distributed over the 12 months of the survey. Residents aged ten years or over from all households drawn for assessment of personal food consumption (n = 34,003) completed two 24-hour food records, on non-consecutive days. In these records, people reported all the foods consumed, the type of preparation and the quantities consumed expressed in the form of household measures. Individual data on date of birth, gender, education, family income and number of people in the household were collected through questionnaires. The list of sociodemographic data includes the location of the household by Federative Unit (UF) and macro-region of Brazil.

In POF 2008–2009, the amounts of food reported in the form of household measures were converted to grams based on the Table of Referenced Measures for Foods Consumed in Brazil^10^ and then converted to energy based on the Table of Nutritional Composition of Foods Consumed in Brazil^11^. For the purpose of this study, culinary preparations were broken down into food and culinary ingredients according to standardized recipes^12^.

### Food Carbon Footprint Coefficients

To estimate the carbon footprint of food consumption reported by the people studied in the POF 2008–2009, coefficients were used that quantify the atmospheric emissions of greenhouse gases, expressed in grams of carbon dioxide equivalent per amount of food consumed (gCO2e/kg)^12^.

Carbon footprint coefficients used in this study are those described in the publication “Footprints of food and culinary preparations consumed in Brazil”^14^. This publication presents, for each food item reported by the people studied by the POF 2008–2009, average environmental impact coefficients calculated based on estimates by studies published in scientific articles or used in environmental product performance reports, and adopts food coefficients similar in the case of foods that did not have available estimates. In the case of culinary preparations, the coefficients consider all the ingredients included in the preparation. Coefficients also consider conversion factors and cooking indices that take into account, respectively, the removal of inedible parts and the incorporation or loss of water due to the cooking effect.

### Data Analysis

The carbon footprint of the daily food consumption of each person studied by the POF 2008–2009 was calculated by adding the products of the amount consumed of each item by its respective carbon footprint coefficient, using the data reported in the two days of the 24-hour food record. Means with 95% confidence intervals of the carbon footprint of daily food consumption (gCO2e/kg/person/day) were calculated for the entire Brazilian population and for sociodemographic strata of this population. These strata were formed based on the location of the household (macro-region and FU) and on the following individual characteristics: gender (male/female), age (10 to 19 years old, 20 to 29, 30 to 39, 40 to 49, 50 to 59, and ≥ 60 years old), fifth of *per capita* family income and years of schooling (≤ 4, 5 to 8, 9 to 12, > 12).

To consider differences between sociodemographic strata regarding the amount of energy consumed, the total daily caloric consumption of each person and the carbon footprint of their diet were calculated at 2,000kcal (gCO_2_e/2,000kcal), and the same analysis made with respect to the carbon footprint was repeated below, without adjustment for the total calories.

Linear regression models were used to test differences between sociodemographic strata of the population in daily caloric intake and in the environmental footprint of the diet. Linear trend tests were used for ordinal categorical variables. For non-ordinal categorical variables or variables without significant linear trend, Bonferroni tests were applied.

All analyses were performed using the *survey* module of the Stata/SE software version 14.0, which considers the effects of complex sampling, allowing for the extrapolation of results to the Brazilian population. The identification of statistical significance was p-value ≤ 0.05.

## RESULTS

Table 1 presents estimates of daily calorie intake for the Brazilian population aged 10 years and over and for sociodemographic strata of this population. An average caloric intake of 1,900kcal/person/day was measured for the population as a whole. In addition, it was found this average was higher among men than among women, tended to decrease with age and increase with income and education, and was higher in the North, intermediate in the Northeast, South and Southeast regions, and lower in the Midwest region.

**Table 1:** Daily food consumption according to sociodemographic variables. Brazilian population aged 10 and over, 2008 to 2009 (n = 34,003).

Variables		Sample distribution (%)	Food consumption (Kcal/person/day)	
			Average	CI95%
Sex				
	Female	52	1,713	(1,696–1,731)
	Male	48	2,102	(2,062–2,143)^a^
Age (years)				
	10 to 19	22	2,010	(1,974–2,046)
	20 to 29	21	2,006	(1,973–2,039)
	30 to 39	18	1,933	(1,899–1,967)
	40 to 49	16	1,852	(1,813–1,890)
	50 to 59	12	1,778	(1,723–1,832)
	≥ 60	13	1,683	(1,570–1,796)^a^
Family income per capita (R$)				
	< 225.28	20	1,785	(1,747–1,823)
	225.28–399.75	20	1,922	(1,875–1,970)
	399.76–637.23	20	1,870	(1,830–1,911)
	637.24–1,151.49	20	1,936	(1,896–1,976)
	≥ 1,151.50	20	1,988	(1,904–2,071)^a^
Education (years of study)				
	≤ 4 years	33	1,775	(1,746–1,804)
	5 to 8 years	27	1,925	(1,895–1,957)
	9 to 12 years	30	1,985	(1,956–2,015)
	> 12 years	11	1,990	(1,848–2,130)^a^
Macro-region				
	North	8	2,058	(2,006–2,110)^d^
	Northeast	28	1,944	(1,882–2,007)c,d
	Southeast	43	1,860	(1,826–1,894)b,c
	South	15	1,900	(1,851–1,949)b,c
	Midwest	7	1,806	(1,762–1,850)^b^
**Total**		**100**	**1,900**	**(1,876 –1,924)**

CI95%: 95% Confidence Interval.

a p < 0.05 for dichotomous variables and p for linear trend < 0.05 in the case of ordinal variables.

b,c,d,e p < 0.05 in the Bonferroni test for two-by-two comparisons of macroregions and when macroregions do not share the same superscript letter.

Table 2 presents estimates of the Brazilian population's dietary carbon footprint and sociodemographic strata of this population.

**Table 2:** Carbon footprint of food consumption according to sociodemographic variables. Brazilian population aged 10 and over, 2008 to 2009 (n = 34,003).

Variables		Carbon footprint			
		(gCO_2_e/person/day)		(gCO_2_e/2,000 kcal)	
		Average	CI95%	Average	CI95%
Sex					
	Female	3,934	3,859–4,009	4,641	4,567–4,716
	Male	5,089	4,974–5,205^a^	4,899	4,812–4,986^a^
Age (years)					
	10 to 19	4,369	4,212–4,525	4,355	4,233–4,477
	20 to 29	4,787	4,651–4,923	4,802	4,687–4,917
	30 to 39	4,745	4,599–4,892	4,941	4,805–5,077
	40 to 49	4,627	4,441–4,813	4,981	4,842–5,120
	50 to 59	4,269	4,116–4,421	4,871	4,719–5,023
	60 _+_	3,915	3,751–4,078	4,785	4,630–4,941
Family income per capita (R$)					
	< 225.28	3,901	3,755–4,048	4,338	4,204–4,472
	225.28–399.75	4,431	4,228–4,634	4,650	4,493–4,807
	399.76–637.23	4,432	4,240–4,624	4,771	4,616–4,927
	637.24–1,151.49)	4,746	4,547–4,945	4,931	4,760–5,103
	≥ 1,151.50	4,933	4,767–5,100^a^	5,133	4,986–5,280^a^
Education (years of study)					
	≤ 4 years	4,144	4,024–4,264	4,656	4,551–4,761
	5 to 8 years	4,439	4,308–4,569	4,652	4,542–4,763
	9 to 12 years	4,775	4,657–4,893	4,850	4,745–4,954
	> 12 years	4,890	4,691–5,089^a^	5,145	4,962–5,328^a^
Macro-region					
	North	5,245	5,051–5,439^c^	5,173	5,023–5,324
	Northeast	4,435	4,297–4,573^b^	4646	4,537–4,755^b^
	Southeast	4,281	4,130–4,431^b^	4,623	4,489–4,758^b^
	South	4,510	4,312–4,707^b^	4,738	4,597–4,880^b^
	Midwest	5,052	4,864–5,240^c^	5,641	5,471–5,812
**Total**		**4,489**	**4,407–4,572**	**4,765**	**4,695–4,836**

gCO2e, grams of carbon dioxide equivalent.

CI95%: 95% Confidence Interval.

a p < 0.05 for dichotomous variables and p for linear trend < 0.05 in the case of ordinal variables.

b,c,d,e p < 0.05 in the Bonferroni test for two-by-two comparisons of macroregions and when macroregions do not share the same superscript letter.

**Table 3:** Carbon footprint of food consumption according to Federative Units. Brazilian population aged 10 and over, 2008 to 2009 (n = 34,003).

Federative Unit	Carbon footprint			
	(gCO_2_e/person/day)		gCO_2_e/2,000kcal	
	Average	CI95%	Average	CI95%
Acre	5,673	4,887–6,458	5,719	5,232–6,207
Amapá	5,219	3,881–6,557	5,383	4,203–6,562
Amazonas	4,390	4,100–4,680	4,270	4,045–4,495
Pará	5,556	5,240–5,872	5,328	5,079–5,576
Rondônia	4,733	4,385–5,080	5,123	4,781–5,465
Roraima	5,319	4,472–6,166	5,582	5,004–6,160
Tocantins	6,133	5,338–6,929	6,205	5,725–6,684
Alagoas	3,522	3,212–3,833	4,248	3,969–4,527
Bahia	4,781	4,386–5,177	4,819	4,552–5,086
Ceará	3,812	3,538–4,087	4,312	4,029–4,595
Maranhão	4,506	4,260–4,751	5,357	5,021–5,693
Paraíba	4,025	3,633–4,416	4,575	4,177–4,973
Pernambuco	4,655	4,373–4,936	4,398	4,190–4,605
Piauí	4,967	4,655–5,278	5,185	4,875–5,494
Rio Grande do Norte	4,049	3,786–4,313	4,056	3,842–4,269
Sergipe	5,331	4,872–5,790	4,616	4,174–5,059
Paraná	4,763	4,412–5,115	4,919	4,661–5,178
Rio Grande Do Sul	4,319	4,043–4,595	4,654	4,426–4,883
Santa Catarina	4,442	4,024–4,861	4,595	4,390–4,800
Espírito Santo	4,243	3,883–4,603	4,661	4,354–4,968
Minas Gerais	4,007	3,822–4,192	4,288	4,114–4,463
Rio de Janeiro	4,171	3,903–4,439	4,418	4,166–4,670
São Paulo	4,456	4,201–4,711	4,860	4,636–5,083
Distrito Federal	4,303	3,786–4,820	4,702	4,285–5,118
Goiás	4,912	4,638–5,186	5,875	5,593–6,156
Mato Grosso	5,482	5,051–5,914	5,806	5,483–6,130
Mato Grosso do Sul	5,598	5,300–5,896	5,745	5,505–5,985

95%CI: 95% confidence interval.

The average carbon footprint of the Brazilian diet, of 4,489gCO2e/person/day, was greater among men than among women; showed a curvilinear relationship with age, being maximum between 20 and 49 years; tended to increase with income and with schooling, and was higher in the North and Midwest regions than in other regions of the country. Adjusting for quantitative differences in food consumption, obtained by fixing consumption at 2,000kcal per person, does not substantially change the relationship between sociodemographic variables and the dietary carbon footprint, except for a reduction in the relative excess footprint among men, which remains significant, and by the increase in the relative excess of the diet footprint in the Midwest region, which becomes the region with the largest carbon footprint, surpassing the North region.

The Figure depicts the spatial distribution of the carbon footprint of crude diets and diets adjusted for a fixed consumption of 2,000kcal per person, in the 27 Federative Units of Brazil. The smallest carbon footprints were found in Alagoas (3,522gCO_2_e, gross footprint) and Rio Grande do Norte (4,056gCO_2_e, footprint per 2,000kcal), while the largest were recorded in Tocantins (61,332gCO_2_e, gross footprint; and 6,205gCO_2_e, footprint per 2,000kcal), as shown in Table 4.

**Figure:**
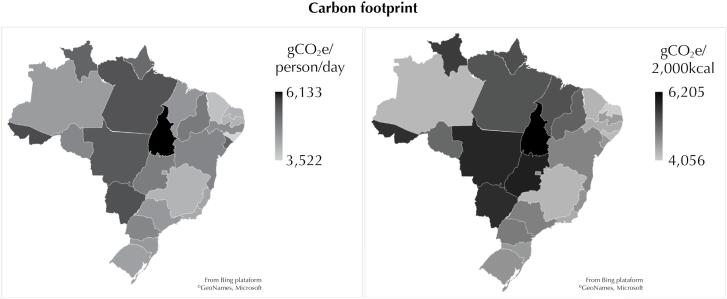
Choropletic maps of mean values of the carbon footprint of food consumption according to Federal Units. Brazilian population aged 10 and over, 2008 to 2009 (n = 34,003).

## DISCUSSION

Based on data from two 24-hour dietary records obtained in 2008 and 2009 from a probabilistic sample of the Brazilian population aged 10 years and over (n = 34,003), the average carbon footprint of the Brazilian diet was estimated at 4,489gCO2e_/_person/day. This footprint was greater in the diet of men, people between 20 and 49 years of age, residents of the North and Midwest regions, and people with a higher level of income or education. Adjusting for differences in the amount of food consumed did not substantially change the relationship of the diet footprint with sociodemographic variables, except for a reduction in the relative excess of the footprint among men and an increase in the relative excess of the footprint in the Midwest region, which would then be the diet with the largest carbon footprint in the country.

When compared to other countries, the carbon footprint of the diet per person/day estimated for Brazil (4,489gCO_2_e) can be considered of intermediate intensity, as it is much higher than that estimated in Peru^13^ (2,036gCO_2_ e), slightly higher than that ofFrance^14^ (4,090gCO_2_e), slightly lower than that of the United States^15^ (4,700gCO_2_e) and much lower than that of Argentina^6^ (5,480gCO_2_e). The carbon footprint of the Brazilian diet exceeds by 30% the value of 3288gCO_2_e/person/day, which corresponds to the estimated footprint of a diet that simultaneously fulfills all the nutritional requirements of a healthy diet^16^ and corroborates with the containment of average temperature of the planet^17^.

The relative excess of the dietary carbon footprint of male representatives observed in our study has also been described in several countries, such as the Netherlands^18^, Ireland^19^ and China^20^. In the Swedish population, similarly to what we found in Brazil, diets with larger carbon footprints were observed among young and intermediate-age adults^21^. The literature does not show a clear pattern of the relationship between the diet's carbon footprint and people's income or education level. For example, in the United States^15^ no differences were observed in the carbon footprint of diets according to socioeconomic variations. In Ireland^19^, the largest carbon footprint was found in the diet of people with intermediate education, while in Sweden^21^, as in Brazil, people with university education had the diets with the largest carbon footprint.

It is worth mentioning that, in Brazil, the higher the level of education, the greater the concern with climate risks^22^. However, this concern was apparently not reflected in the diet's carbon footprint. Relatively low environmental impacts observed in the diets of developing countries have been attributed to the low purchasing power of the population and the consequent restriction to purchase foods with greater environmental impact, such as meat^13^. A study comparing higher and lower income Peruvian populations concluded that in the city of Lima, where the population's purchasing power is greater, it would be possible to reduce the carbon footprint of the diet by 6% without compromising its nutritional quality, while in Cajamarca, where there are the poorest, the improvement in the nutritional quality of the diet would lead to an 18% increase in its carbon footprint^13^.

The largest carbon footprints of the Brazilian diet were found in the North and Midwest regions. Although it is beyond the scope of this study to analyze the impact of specific foods on the carbon footprint of the Brazilian diet, which will be the subject of a future study, it should be noted that, in the two regions where the carbon footprint of the diet is higher the consumption of beef is also higher: 58.6g/person/day in the North region and 80.7g/person/day in the Midwest region, consumption much higher than the average consumption recorded in Brazil, which is 50.2g /person/day^9^.

A previous study carried out with the same database estimated the carbon footprint of the Brazilian diet at 6,761gCO_2_/person/day^23^, therefore, about 50% higher than our estimate. It should be noted that this study considered only adults and excluded foods and beverages that did not have an environmental footprint, which represented about 15% of the total calories consumed. In addition, it did not take into account the form of food consumption, which can lead to errors and inconsistencies in estimating the environmental impact of diets^24^. For example, the carbon footprint of cooked rice, one of the most consumed foods in Brazil, is 2.3 times smaller than the footprint of an equivalent amount of raw rice^12^.

Another important difference between the two studies, which is probably the most important to explain the difference found regarding the magnitude of the carbon footprint of the Brazilian diet, concerns the coefficient used to quantify the carbon footprint of beef: 60kgCO2e/kg in the previous study and 26.3kgCO_2_e/kg in our study. The coefficient used in our study corresponded to the average of values found in the international literature and was close to the coefficient calculated by Clune et al.^25^ and used in studies carried out in Argentina^6^ and Peru^13^ (28kgCO_2_e/kg), while the coefficient used in the previous study was calculated based on a single study that considered zootechnical parameters of extensive cattle raising and emissions from pastures^26^, which have low feed conversion and high methane emissions.

Among the limitations of our study, we highlight the fact that, although we used estimated environmental impact coefficients for foods and culinary preparations reported by POF 2008-2009^9^ participants, due to the scarcity of studies carried out in Brazil, the estimates were often based on studies carried out in other countries. Another important limitation of this study is due to the fact that the coefficients for many industrialized foods come not from published studies, but from statements of environmental performance of products^12^.

The strengths of our study are linked to the representativeness of the studied sample and the methodological procedures adopted. Adjusting the environmental footprint coefficients according to how food is consumed reduced the possibilities of errors and inconsistencies^23^. In addition, the calculation of the environmental impacts of diets by fixed amounts of calories provided an adequate comparison between dietary patterns by controlling the effects of differences in the amount of food consumed.

## CONCLUSION

The carbon footprint of the Brazilian diet, estimated by our study at 4,489gCO2e/person/day, exceeds by about 30% the human diet footprint that could simultaneously meet the nutritional requirements of a healthy diet and the global target to contain the increase of the planet's average temperature. The pattern of association between sociodemographic variables and dietary carbon footprints described in this study can help identify priority targets for public actions aimed at reducing the environmental impacts of food consumption in Brazil.

## References

[1] Food and Agriculture Organization of the United Nations; World Health Organization. Sustainable healthy diets: guiding principles. Rome (IT): FAO; WHO; 2019 [cited 2020 Nov 26]. Available from: http://www.fao.org/3/ca6640en/ca6640en.pdf

[2] Objetivos de Desenvolvimento Sustentável. Brasília, DF: Instituto de Pesquisa Econômica Aplicada; 2019 [cited 2020 Nov 26]. Available from: https://www.ipea.gov.br/ods/index.html

[3] The Giessen Declaration. Public Health Nutr. 2005 [cited 2020 Nov 26];8(6A):783-6. Available from: http://archive.wphna.org/wp-content/uploads/2013/03/05-PHN-8-6A-The-Giessen-Declaration.pdf 10.1079/phn2005768 16236217

[4] Associação Brasileira de Normas Técnicas. NBR ISO 14040. Gestão ambiental - Avaliação do ciclo de vida - Princípios e estrutura. Rio de Janeiro: ABNT; 2001.

[5] Tilman D, Clark M. Global diets link environmental sustainability and human health. Nature. 2014;515(7528):518-22. https://doi.org/10.1038/nature13959 10.1038/nature13959 25383533

[6] Arrieta EM, González AD. Impact of current, National Dietary Guidelines and alternative diets on greenhouse gas emissions in Argentina. Food Policy. 2018;79:58-66. https://doi.org/10.1016/j.foodpol.2018.05.003 10.1016/j.foodpol.2018.05.003

[7] Batlle-Bayer L, Bala A, García-Herrero I, Lemaire E, Song G, Aldaco R, et al. The Spanish Dietary Guidelines: a potential tool to reduce greenhouse gas emissions of current dietary patterns. J Clean Prod. 2019;213:588-98. https://doi.org/10.1016/j.jclepro.2018.12.215 10.1016/j.jclepro.2018.12.215

[8] Grosso G, Fresán U, Bes-Rastrollo M, Marventano S, Galvano F. Environmental impact of dietary choices: role of the Mediterranean and other dietary patterns in an Italian cohort. Int J Environ Res Public Health. 2020;17(5):1468. https://doi.org/10.3390/ijerph17051468 10.3390/ijerph17051468 PMC7084186 32106472

[9] Instituto Brasileiro de Geografia e Estatística, Diretoria de Pesquisa, Coordenação de Trabalho e Rendimento. Pesquisa de Orçamentos Familiares 2008 - 2009: análise do consumo alimentar pessoal no Brasil. Rio de Janeiro: IBGE; 2011.

[10] Instituto Brasileiro de Geografia e Estatística, Diretoria de Pesquisa, Coordenação de Trabalho e Rendimento. Pesquisa de Orçamentos Familiares 2008-2009: tabela de medidas referidas para os alimentos consumidos no Brasil. Rio de Janeiro: IBGE; 2011.

[11] Instituto Brasileiro de Geografia e Estatística, Diretoria de Pesquisa, Coordenação de Trabalho e Rendimento. Pesquisa de Orçamentos Familiares 2008-2009: tabelas de composição nutricional dos alimentos consumidos no Brasil. Rio de Janeiro: IBGE; 2011.

[12] Garzillo JMF, Machado PP, Louzada MLC, Levy RB, Monteiro, CA. Pegadas dos alimentos e das preparações culinárias consumidos no Brasil. São Paulo: Faculdade de Saúde Pública da USP; 2019 [citado 19 fev 2021]. (e-Coleções FSP/USP). Disponível em: http://colecoes.sibi.usp.br/fsp/items/show/3592

[13] Larrea-Gallegos G, Vázquez-Rowe I. Optimization of the environmental performance of food diets in Peru combining linear programming and life cycle methods. Sci Total Environ. 2020;699:134231. https://doi.org/10.1016/j.scitotenv.2019.134231 10.1016/j.scitotenv.2019.134231 31677472

[14] Vieux F, Darmon N, Touazi D, Soler LG. Greenhouse gas emissions of self-selected individual diets in France: changing the diet structure or consuming less? Ecol Econ. 2012;75:91-101. https://doi.org/10.1016/j.ecolecon.2012.01.003 10.1016/j.ecolecon.2012.01.003

[15] Heller MC, Willits-Smith A, Meyer R, Keoleian GA, Rose D. Greenhouse gas emissions and energy use associated with production of individual self-selected US diets. Environ Res Lett. 2018;13(4):044004. https://doi.org/10.1088/1748-9326/aab0ac 10.1088/1748-9326/aab0ac PMC5964346 29853988

[16] World Health Organization. Healthy diet. Fact Sheet; nº 394. Geneva (CH): WHO; updated 2015 [citado 19 fev 2021].. Disponível em: https://www.who.int/nutrition/publications/nutrientrequirements/healthydiet_factsheet394.pdf

[17] Ritchie H, Reay DS, Higgins P. The impact of global dietary guidelines on climate change. Glob Environ Change. 2018;49:46-55. https://doi.org/10.1016/j.gloenvcha.2018.02.005 10.1016/j.gloenvcha.2018.02.005

[18] Temme EHM, Toxopeus IB, Kramer GFH, Brosens MCC, Drijvers JMM, Tyszler M, et al. Greenhouse gas emission of diets in the Netherlands and associations with food, energy and macronutrient intakes. Public Health Nutr. 2015;18(13):2433-45. https://doi.org/10.1017/S1368980014002821 10.1017/S1368980014002821 PMC10271514 25543460

[19] Hyland JJ, Henchion M, McCarthy M, McCarthy SN. The climatic impact of food consumption in a representative sample of Irish adults and implications for food and nutrition policy. Public Health Nutr. 2017;20(4):726-38. https://doi.org/10.1017/S1368980016002573 10.1017/S1368980016002573 PMC10261633 27667716

[20] Song G, Li M, Fullana-i-Palmer P, WilliamsonD, Wang Y. Dietary changes to mitigate climate change and benefit public health in China. Sci Total Environ. 2017;577:289-98. https://doi.org/10.1016/j.scitotenv.2016.10.184 10.1016/j.scitotenv.2016.10.184 27802883

[21] Strid A, Hallström E, Hjorth T, Johansson I, Lindahl B, Sonesson U, et al. Climate impact from diet in relation to background and sociodemographic characteristics in the Västerbotten Intervention Program. Public Health Nutr. 2019;22(17):3288-97. https://doi.org/10.1017/S1368980019002131 10.1017/S1368980019002131 PMC10260555 31566152

[22] Bursztyn M, Eiró F. Mudanças climáticas e distribuição social da percepção de risco no Brasil. Soc Estado. 2015;30(2):471-93. https://doi.org/10.1590/S0102-699220150002000010 10.1590/S0102-699220150002000010

[23] Travassos GF, Cunha DA, Coelho AB. The environmental impact of Brazilian adults’ diet. J Clean Prod. 2020;272:122622. https://doi.org/10.1016/j.jclepro.2020.122622 10.1016/j.jclepro.2020.122622

[24] Heller MC, Keoleian GA, Willet WC. Toward a life cycle-based, diet-level framework for food environmental impact and nutrition quality assessment: a critical review. Environ Sci Technol. 2013;47(22):12632-47. https://doi.org/10.1021/es4025113 10.1021/es4025113 24152032

[25] Clune S, Crossin E, Verghese K. Systematic review of greenhouse gas emissions for different fresh food categories. J Clean Prod. 2017;140 (Part 2):766-83. https://doi.org/10.1016/j.jclepro.2016.04.082 10.1016/j.jclepro.2016.04.082

[26] Blonk H, Kool A, Luske B, De Waart S. Environmental effects of protein-rich food products in the Netherlands: consequences of animal protein substitutes. Gouda (NL): Blonk Consulktants; 2008. Disponível em http://www.blonkconsultants.nl/wp-content/uploads/2016/06/english-summary-protein-rich-products.pdf

