# Supplementary material for: Carbon footprint of the Brazilian diet
Source: Rev Saude Publica. 2021 Nov 23;55:90. doi: 10.11606/s1518-8787.2021055003614 (PMC8621484; doi:10.11606/s1518-8787.2021055003614)
Supplement: Translation

# Translation: Pegada de carbono da dieta no Brasil

**Authors:** Josefa Maria Fellegger Garzillo, Priscila Pereira Machado, Fernanda Helena Marrocos Leite, Euridice Martinez Steele, Vanessa Fadanelli Schoenardie Poli, Maria Laura da Costa Louzada, Renata Bertazzi Levy, Carlos Augusto Monteiro

**Affiliations:** I São Paulo SP Brasil Universidade de São Paulo. Núcleo de Pesquisas Epidemiológicas em Nutrição e Saúde. São Paulo, SP, Brasil; II Melbourne Austrália Deakin University. Institute for Physical Activity and Nutrition. Melbourne, Austrália; III São Paulo SP Brasil Universidade de São Paulo. Faculdade de Medicina. Departamento de Medicina Preventiva. São Paulo, SP, Brasil

**Keywords:** Pegada de Carbono, Alimentação Básica, Fatores Socioeconômicos, Brasil

## Abstract

**OBJETIVO:**

Estimar a pegada de carbono da dieta brasileira e de estratos sociodemográficos dessa população.

**MÉTODOS:**

A pegada de carbono da dieta foi estimada com base nos dados de dois registros alimentares de 24 horas, obtidos em 2008 e 2009, de uma amostra probabilística da população brasileira com 10 ou mais anos de idade (n = 34.003) e em coeficientes de impacto ambiental de alimentos e preparações culinárias consumidos no Brasil (gCO2e/kg). Médias com intervalos de confiança de 95% do consumo alimentar (kcal/pessoa/dia) e da pegada de carbono da dieta (gCO2e/pessoa/dia e em gCO2e/2.000kcal) foram calculadas para o conjunto da população e para estratos segundo sexo, idade, renda, escolaridade, macrorregiões e Unidade Federativa. Modelos de regressão linear foram utilizados para identificar diferenças significativas (p < 0,05) na pegada de carbono da dieta de diferentes estratos sociodemográficos.

**RESULTADOS:**

A pegada média de carbono da dieta brasileira foi de 4.489gCO2e/pessoa/dia. Foi maior para o sexo masculino, para a faixa etária de 20 a 49 anos e para as regiões Norte e Centro-Oeste, e tendeu a aumentar com a renda e a escolaridade. O padrão de associação da pegada a variáveis sociodemográficas não se alterou substancialmente com o ajuste para diferenças na quantidade consumida de alimentos, exceto por uma redução no excesso relativo da pegada entre homens e pelo aumento no excesso relativo da pegada na região Centro-Oeste.

**CONCLUSÃO:**

A pegada de carbono da dieta brasileira excede em cerca de 30% a pegada da dieta humana que poderia atender, simultaneamente, os requisitos nutricionais de uma dieta saudável e a meta global de contenção do aumento da temperatura média do planeta. O padrão de associação dessa pegada às variáveis sociodemográficas pode auxiliar na identificação de alvos prioritários para ações públicas que visem a reduzir os impactos ambientais do consumo alimentar no Brasil.

## INTRODUÇÃO

A transição para um sistema alimentar sustentável é uma urgência coletiva, haja vista a gravidade das mudanças ambientais globais e os impactos da alimentação no equilíbrio ecológico^1^. O Brasil assumiu compromissos multilaterais como a eliminação da fome e o combate às mudanças climáticas^2^, o que demanda das ciências nutricional e ambiental a união de esforços para compreender os múltiplos impactos da alimentação^3^ e construir diretrizes sobre dietas adequadas, saudáveis e sustentáveis nas condições brasileiras, esclarecendo a população e os governos sobre como proteger a natureza^1^.

Os estudos dos impactos ou das pegadas ambientais da alimentação requerem a disponibilidade de dados representativos do consumo alimentar da população e indicadores que quantifiquem as pegadas dos alimentos individuais que compõem a alimentação. Esses indicadores, calculados com a metodologia de avaliação do ciclo de vida de produtos^4^, contabilizam o uso dos recursos naturais e a carga de poluentes lançados no meio ambiente por quilo de alimento, como o coeficiente da pegada de carbono do alimento, que quantifica a quantidade de emissões atmosféricas de gases do efeito estufa. Com base em estudos do impacto ambiental da alimentação, padrões alimentares como a “dieta mediterrânea” e a “dieta vegetariana” são considerados modelos para a mitigação dos efeitos negativos da alimentação sobre o ambiente^5-8^.

As avaliações do impacto ambiental das dietas que abrangem a população em geral, bem como estratos sociodemográficos específicos, são relevantes porque identificam tanto os pontos críticos a se mudar na alimentação, quanto os grupos populacionais nos quais as políticas públicas devem focar para reduções substanciais dos impactos ambientais.

Neste artigo, com base em dados coletados em inquérito nacional sobre o consumo de alimentos no Brasil, estimamos a pegada de carbono da dieta consumida pela população brasileira e da dieta de estratos sociodemográficos dessa população.

## MÉTODOS

### Fonte dos Dados sobre Consumo Alimentar

Os dados sobre consumo alimentar analisados neste estudo são provenientes do módulo de avaliação do consumo alimentar pessoal da Pesquisa de Orçamentos Familiares (POF) realizada pelo Instituto Brasileiro de Geografia e Estatística (IBGE) entre maio de 2008 e maio de 2009 (POF 2008–2009)^9^.

A POF 2008–2009 empregou plano amostral complexo, por conglomerados, com estratificação geográfica e socioeconômica de todos os setores censitários do país, seguida de sorteios aleatórios de setores em um primeiro estágio e de domicílios em um segundo. O número de setores sorteados em cada estrato foi proporcional ao número de domicílios no estrato. O sorteio dos domicílios de cada setor foi feito por amostragem aleatória simples sem reposição. A amostra abrangeu 55.970 domicílios e o módulo de avaliação do consumo alimentar pessoal foi aplicado em uma subamostra aleatória de 13.569 domicílios (24,3% do total de domicílios estudados)^9^.

As entrevistas realizadas pela POF 2008–2009 em cada estrato da amostra foram distribuídas uniformemente ao longo dos 12 meses de realização da pesquisa. Os moradores com dez ou mais anos de idade de todos os domicílios sorteados para a avaliação do consumo alimentar pessoal (n = 34.003) preencheram dois registros alimentares de 24 horas, em dias não consecutivos. Nesses registros, as pessoas relatavam todos os alimentos consumidos, o tipo de preparação e as quantidades consumidas expressas na forma de medidas caseiras. Dados individuais sobre data de nascimento, sexo, escolaridade, renda familiar e número de pessoas no domicílio foram coletados por meio de questionários. O rol de dados sociodemográficos contempla a localização do domicílio por Unidade Federativa (UF) e macrorregião do Brasil.

Na POF 2008–2009, as quantidades de alimentos relatadas na forma de medidas caseiras foram convertidas em gramas com base na Tabela de Medidas Referidas para os Alimentos Consumidos no Brasil^10^ e, em seguida, convertidas em energia com base na Tabela de Composição Nutricional dos Alimentos Consumidos no Brasil^11^. Para efeito do presente estudo, preparações culinárias foram fragmentadas em alimentos e ingredientes culinários conforme receitas padronizadas^12^.

### Coeficientes da Pegada de Carbono de Alimentos

Para estimar a pegada de carbono do consumo alimentar informado pelas pessoas estudadas na POF 2008–2009, foram usados coeficientes que quantificam as emissões atmosféricas de gases do efeito estufa, expressas em gramas de dióxido de carbono equivalente por quantidade de alimento consumido (gCO2e/kg)^12^.

Os coeficientes da pegada de carbono utilizados neste estudo são aqueles descritos na publicação “Pegadas dos alimentos e preparações culinárias consumidos no Brasil”^14^. Essa publicação apresenta, para cada item alimentar relatado pelas pessoas estudadas pela POF 2008–2009, coeficientes de impacto ambiental médios calculados com base em coeficientes estimados por estudos publicados em artigos científicos ou utilizados em relatórios de desempenho ambiental de produtos, e adota coeficientes de alimentos similares no caso de alimentos que não contavam com estimativas disponíveis. No caso de preparações culinárias, os coeficientes levam em conta todos os ingredientes incluídos na preparação. Os coeficientes consideram, ainda, fatores de conversão e índices de cocção que levam em conta, respectivamente, a retirada de partes não comestíveis e a incorporação ou perda de água pelo efeito do cozimento.

### Análise dos Dados

A pegada de carbono do consumo alimentar diário de cada pessoa estudada pela POF 2008–2009 foi calculada somando os produtos da quantidade consumida de cada item pelo seu respectivo coeficiente de pegada de carbono, utilizando os dados informados nos dois dias de registro alimentar de 24 horas. Médias com intervalos de confiança de 95% da pegada de carbono do consumo alimentar diário (gCO2e/kg/pessoa/dia) foram calculadas para o conjunto da população brasileira e para estratos sociodemográficos dessa população. Esses estratos foram constituídos com base na localização do domicílio (macrorregião e UF) e nas seguintes características individuais: sexo (masculino/feminino), idade (10 a 19 anos, 20 a 29, 30 a 39, 40 a 49, 50 a 59 e ≥ 60 anos), quinto da renda familiar *per capita* e anos de escolaridade (≤ 4, 5 a 8, 9 a 12, > 12).

Visando levar em conta diferenças entre os estratos sociodemográficos quanto à quantidade de energia consumida, calculou-se o consumo calórico diário total de cada pessoa e a pegada de carbono da sua alimentação fixada em 2.000kcal (gCO2e/2.000kcal), e repetiu-se, a seguir, a mesma análise feita com relação à pegada de carbono sem ajuste para o total calórico.

Modelos de regressão linear foram utilizados para testar diferenças entre estratos sociodemográficos da população no consumo calórico diário e nas pegadas ambientais da dieta. Testes de tendência linear foram utilizados para variáveis categóricas ordinais. Para variáveis categóricas não ordinais ou variáveis sem tendência linear significativa, foram aplicados testes de Bonferroni.

Todas as análises foram realizadas no módulo *survey* do software Stata/SE versão 14.0, que considera os efeitos da amostragem complexa permitindo a extrapolação dos resultados para a população brasileira. A identificação das significâncias estatísticas foi valor-p ≤ 0,05.

## RESULTADOS

A Tabela 1 apresenta estimativas da ingestão diária de calorias para a população brasileira com 10 ou mais anos de idade e para estratos sociodemográficos dessa população. Aferiu-se uma ingestão calórica média de 1.900kcal/pessoa/dia para o conjunto da população. Além disso, constatou-se que essa média foi maior entre homens do que entre mulheres, tendeu a diminuir com a idade e a aumentar com a renda e com a escolaridade, e foi maior na região Norte, intermediária nas regiões Nordeste, Sul e Sudeste e menor na região Centro-Oeste.

**Tabela 1:** Consumo alimentar diário segundo variáveis sociodemográficas. População brasileira com 10 ou mais anos de idade, 2008 a 2009 (n = 34.003).

Variáveis		Distribuição da amostra (%)	Consumo alimentar (Kcal/pessoa/dia)	
			Média	IC95%
Sexo				
	Feminino	52	1.713	(1.696–1.731)
	Masculino	48	2.102	(2.062–2.143)^a^
Idade (anos)				
	10 a 19	22	2.010	(1.974–2.046)
	20 a 29	21	2.006	(1.973–2.039)
	30 a 39	18	1.933	(1.899–1.967)
	40 a 49	16	1.852	(1.813–1.890)
	50 a 59	12	1.778	(1.723–1.832)
	≥ 60	13	1.683	(1.570–1.796)^a^
Renda familiar per capita (R$)				
	< 225,28	20	1.785	(1.747–1.823)
	225,28–399,75	20	1.922	(1.875–1.970)
	399,76–637,23	20	1.870	(1.830–1911)
	637,24–1.151,49)	20	1.936	(1.896–1.976)
	≥ 1.151,50	20	1.988	(1.904–2.071)^a^
Escolaridade (anos de estudo)				
	≤ 4 anos	33	1.775	(1.746–1.804)
	5 a 8 anos	27	1.925	(1.895–1.957)
	9 a 12 anos	30	1.985	(1.956–2.015)
	> 12 anos	11	1.990	(1.848–2.130)^a^
Macrorregião				
	Norte	8	2.058	(2.006–2.110)^d^
	Nordeste	28	1.944	(1.882–2.007)c,d
	Sudeste	43	1.860	(1.826–1.894)b,c
	Sul	15	1.900	(1.851–1.949)b,c
	Centro-Oeste	7	1.806	(1.762–1.850)^b^
**Total**		**100**	**1.900**	**(1.876–1924)**

IC95%: intervalo de confiança de 95%.

a p < 0,05 para variáveis dicotômicas e p para tendência linear < 0,05 no caso de variáveis ordinais.

b,c,d,e p < 0,05 no teste de Bonferroni para comparações das macrorregiões duas a duas e quando as macrorregiões não compartilham a mesma letra em sobrescrito.

A Tabela 2 apresenta estimativas da pegada de carbono da dieta da população brasileira e de estratos sociodemográficos dessa população.

**Tabela 2:** Pegada de carbono do consumo alimentar segundo variáveis sociodemográficas. População brasileira com 10 ou mais anos ou mais de idade, 2008 a 2009 (n = 34.003).

Variáveis		Pegada de carbono			
		(gCO_2_e/pessoa/dia)		(gCO_2_e/2.000 kcal)	
		Média	IC95%	Média	IC95%
Sexo					
	Feminino	3.934	3.859–4.009	4.641	4.567–4.716
	Masculino	5.089	4.974–5.205^a^	4.899	4.812–4.986^a^
Idade (anos)					
	10 a 19	4.369	4.212–4.525	4.355	4.233–4.477
	20 a 29	4.787	4.651–4.923	4.802	4.687–4.917
	30 a 39	4.745	4.599–4.892	4.941	4.805–5.077
	40 a 49	4.627	4.441–4.813	4.981	4.842–5.120
	50 a 59	4.269	4.116–4.421	4.871	4.719–5.023
	60 e _+_	3.915	3.751–4.078	4.785	4.630–4.941
Renda familiar per capita (R$)					
	< 225,28	3.901	3.755–4.048	4.338	4.204–4.472
	225,28–399,75	4.431	4.228–4.634	4.650	4.493–4.807
	399,76–637,23	4.432	4.240–4.624	4.771	4.616–4.927
	637,24–1.151,49)	4.746	4.547–4.945	4.931	4.760–5.103
	≥ 1.151,50	4.933	4.767–5.100^a^	5.133	4.986–5.280^a^
Escolaridade (anos de estudo)					
	≤ 4 anos	4.144	4.024–4.264	4.656	4.551–4.761
	5 a 8 anos	4.439	4.308–4.569	4.652	4.542–4.763
	9 a 12 anos	4.775	4.657–4.893	4.850	4.745–4.954
	> 12 anos	4.890	4.691–5.089^a^	5.145	4.962–5.328^a^
Macrorregião					
	Norte	5.245	5.051–5.439^c^	5.173	5.023–5.324
	Nordeste	4.435	4.297–4.573^b^	4.646	4.537–4.755^b^
	Sudeste	4.281	4.130–4.431^b^	4.623	4.489–4.758^b^
	Sul	4.510	4.312–4.707^b^	4.738	4.597–4.880^b^
	Centro-Oeste	5.052	4.864–5.240^c^	5.641	5.471–5.812
**Total**		**4.489**	**4.407–4.572**	**4.765**	**4.695–4.836**

gCO_2_e, gramas de dióxido de carbono equivalente.

IC95%, intervalo de confiança de 95%.

a p < 0,05 para variáveis dicotômicas e p para tendência linear < 0,05 no caso de variáveis ordinais.

b,c,d,e p < 0,05 no teste de Bonferroni para comparações das macrorregiões duas a duas e quando as macrorregiões não compartilham a mesma letra em sobrescrito.

**Tabela 3:** Pegada de carbono do consumo alimentar segundo as Unidades Federativas. População brasileira com 10 anos ou mais de idade, 2008 a 2009 (n = 34.003).

Unidade Federativa	Pegada de carbono			
	gCO_2_e/pessoa/dia		gCO_2_e/2.000kcal	
	Média	IC95%	Média	IC95%
Acre	5.673	4.887–6.458	5.719	5.232–6.207
Amapá	5.219	3.881–6.557	5.383	4.203–6.562
Amazonas	4.390	4.100–4.680	4.270	4.045–4.495
Pará	5.556	5.240–5.872	5.328	5.079–5.576
Rondônia	4.733	4.385–5.080	5.123	4.781–5.465
Roraima	5.319	4.472–6.166	5.582	5.004–6.160
Tocantins	6.133	5.338–6.929	6.205	5.725–6.684
Alagoas	3.522	3.212–3.833	4.248	3.969–4.527
Bahia	4.781	4.386–5.177	4.819	4.552–5.086
Ceará	3.812	3.538–4.087	4.312	4.029–4.595
Maranhão	4.506	4.260–4.751	5.357	5.021–5.693
Paraíba	4.025	3.633–4.416	4.575	4.177–4.973
Pernambuco	4.655	4.373–4.936	4.398	4.190–4.605
Piauí	4.967	4.655–5.278	5.185	4.875–5.494
Rio Grande do Norte	4.049	3.786–4.313	4.056	3.842–4.269
Sergipe	5.331	4.872–5.790	4.616	4.174–5.059
Paraná	4.763	4.412–5.115	4.919	4.661–5.178
Rio Grande Do Sul	4.319	4.043–4.595	4.654	4.426–4.883
Santa Catarina	4.442	4.024–4.861	4.595	4.390–4.800
Espírito Santo	4.243	3.883–4.603	4.661	4.354–4.968
Minas Gerais	4.007	3.822–4.192	4.288	4.114–4.463
Rio de Janeiro	4.171	3.903–4.439	4.418	4.166–4.670
São Paulo	4.456	4.201–4.711	4.860	4.636–5.083
Distrito Federal	4.303	3.786–4.820	4.702	4.285–5.118
Goiás	4.912	4.638–5.186	5.875	5.593–6.156
Mato Grosso	5.482	5.051–5.914	5.806	5.483–6.130
Mato Grosso do Sul	5.598	5.300–5.896	5.745	5.505–5.985

IC95: intervalo de confiança de 95%.

A pegada de carbono média da dieta brasileira, de 4.489gCO2e/pessoa/dia, foi maior entre homens do que entre mulheres, mostrou uma relação curvilínea com a idade, sendo máxima entre 20 e 49 anos, tendeu a aumentar com a renda e com a escolaridade e foi maior nas regiões Norte e Centro-Oeste do que nas demais regiões do país. O ajuste para diferenças quantitativas no consumo alimentar, obtido fixando o consumo de 2.000kcal por pessoa, não modifica substancialmente a relação entre variáveis sociodemográficas e a pegada de carbono da dieta, exceto por uma redução no excesso relativo da pegada entre homens, que permanece significativo, e pelo aumento no excesso relativo da pegada da dieta na região Centro-Oeste, que passa a ser a região com a maior pegada de carbono, superando a região Norte.

A Figura retrata a distribuição espacial das pegadas de carbono das dietas brutas e das dietas ajustadas para um consumo fixo de 2.000kcal por pessoa, nas 27 Unidades Federativas do Brasil. As menores pegadas de carbono foram encontradas em Alagoas (3.522gCO_2_e, pegada bruta) e no Rio Grande do Norte (4.056gCO_2_e, pegada por 2.000kcal), enquanto as maiores foram registradas em Tocantins (61.332gCO_2_e, pegada bruta; e 6.205gCO_2_e, pegada por 2.000kcal), conforme a Tabela 4.

**Figura:** Mapas coropléticos de valores médios da pegada de carbono do consumo alimentar segundo Unidades Federativas. População brasileira com 10 ou mais anos de idade, 2008 a 2009 (n = 34.003).

## DISCUSSÃO

Com base em dados de dois registros alimentares de 24 horas obtidos em 2008 e 2009 de amostra probabilística da população brasileira com 10 ou mais anos de idade (n = 34.003), estimou-se a pegada média de carbono da dieta no Brasil em 4.489gCO_2_e/pessoa/dia. Esta pegada foi maior na dieta de homens, de pessoas entre 20 e 49 anos de idade, de residentes nas regiões Norte e Centro-Oeste e de pessoas com maior nível de renda ou de escolaridade. O ajuste para diferenças na quantidade consumida de alimentos não modificou substancialmente a relação da pegada da dieta com variáveis sociodemográficas, exceto por uma redução no excesso relativo da pegada entre homens e pelo aumento no excesso relativo da pegada na região Centro-Oeste, que passou a ter a dieta com maior pegada de carbono no país.

Quando comparada à de outros países, a pegada de carbono da dieta por pessoa/dia que estimamos para o Brasil (4.489gCO_2_e) pode ser considerada de intensidade intermediária, por ser muito maior que a estimada no Peru^13^ (2.036gCO_2_e), ligeiramente maior que a da França^14^ (4.090gCO_2_e), ligeiramente menor que a dos Estados Unidos^15^ (4.700gCO_2_e) e muito menor que a da Argentina^6^ (5.480gCO_2_e). A pegada de carbono da dieta brasileira excede em cerca de 30% o valor de 3288 gCO_2_e/pessoa/dia, que corresponde ao estimado para a pegada de uma dieta que concomitantemente preenche todos os requisitos nutricionais de uma dieta saudável^16^ e corrobora com a contenção da temperatura média do planeta^17^.

O excesso relativo da pegada de carbono da dieta de representantes sexo masculino observado em nosso estudo tem sido descrito também em vários países, como Holanda^18^, Irlanda^19^ e China^20^. Na população sueca, similarmente ao que encontramos no Brasil, as dietas com maiores pegadas de carbono foram observadas entre adultos jovens e de idade intermediária^21^. A literatura não mostra um padrão claro de relação da pegada de carbono da dieta com o nível de renda ou de educação das pessoas. Por exemplo, nos Estados Unidos^15^ não foram observadas diferenças nas pegadas de carbono das dietas conforme variações socioeconômicas. Já na Irlanda^19^, as maiores pegadas de carbono foram encontradas na dieta de pessoas com escolaridade intermediária, enquanto na Suécia^21^, como no Brasil, pessoas com escolaridade universitária apresentaram as dietas com as maiores pegadas de carbono.

Vale mencionar que, no Brasil, quanto maior a escolaridade, maior a preocupação com os riscos climáticos^22^. No entanto, essa preocupação aparentemente não se refletiu na pegada de carbono da dieta. Impactos ambientais relativamente baixos observados nas dietas dos países em desenvolvimento têm sido atribuídos ao baixo poder aquisitivo da população e à consequente restrição para adquirir alimentos com maior impacto ambiental, como a carne^13^. Um estudo que compara populações do Peru com maior ou menor renda concluiu que na cidade de Lima, onde é maior o poder aquisitivo da população, seria possível reduzir a pegada de carbono da dieta em 6% sem prejuízo de sua qualidade nutricional, enquanto em Cajamarca, onde há mais pobres, a melhoria na qualidade nutricional da dieta levaria a um aumento de 18% em sua pegada de carbono^13^.

As maiores pegadas de carbono da dieta brasileira foram encontradas nas regiões Norte e Centro-Oeste. Ainda que fuja do escopo deste estudo a análise do impacto de alimentos específicos sobre a pegada de carbono da dieta brasileira, o que será motivo de um próximo estudo, deve-se notar que, nas duas regiões onde é maior a pegada de carbono da dieta, é também maior o consumo de carne bovina: 58,6g/pessoa/dia na região Norte e 80,7g/pessoa/dia na região Centro-Oeste, consumo bastante superior ao consumo médio registrado no Brasil, que é de 50,2g/pessoa/dia^9^.

Estudo anterior realizado com a mesma base de dados estimou a pegada de carbono da dieta brasileira em 6.761gCO_2_/pessoa/dia^23^, portanto, cerca de 50% maior do que nossa estimativa. Importa notar que esse estudo considerou apenas adultos e excluiu alimentos e bebidas que não dispunham de pegada ambiental, que representaram cerca de 15% do total de calorias consumidas. Além disso, não levou em conta a forma de consumo dos alimentos, o que pode levar a erros e inconsistências na estimativa do impacto ambiental das dietas^24^. Por exemplo, a pegada de carbono do arroz cozido, um dos alimentos mais consumidos no Brasil, é 2,3 vezes menor do que a pegada de uma quantidade equivalente de arroz cru^12^.

Outra diferença importante entre os dois estudos e que é provavelmente a mais importante para explicar a diferença encontrada quanto à magnitude da pegada de carbono da dieta brasileira, diz respeito ao coeficiente utilizado para quantificar a pegada de carbono da carne bovina: 60kgCO2e/kg no estudo anterior e 26,3kgCO_2_e/kg em nosso estudo. O coeficiente utilizado em nosso estudo correspondeu à média de valores encontrados na literatura internacional e ficou próximo ao coeficiente calculado por Clune e colaboradores^25^ e utilizado em estudos realizados na Argentina^6^ e no Peru^13^ (28kgCO_2_e/kg), enquanto o coeficiente utilizado no estudo anterior foi calculado com base em um único estudo que levou em conta parâmetros zootécnicos da pecuária extensiva e emissões das pastagens^26^, que apresentam baixa conversão alimentar e alta emissão de metano.

Dentre as limitações de nosso estudo, destacamos o fato de que, embora tenhamos empregado coeficientes de impacto ambiental estimados para alimentos e preparações culinárias relatados pelos participantes da POF 2008–2009^9^, devido à escassez de estudos realizados no Brasil, as estimativas frequentemente foram baseadas em estudos realizados em outros países. Outra limitação importante deste estudo decorre do fato de os coeficientes relativos a muitos alimentos industrializados procederem não propriamente de estudos publicados, mas de declarações de desempenho ambiental de produtos^12^.

Os pontos fortes de nosso estudo estão ligados à representatividade da amostra estudada e aos procedimentos metodológicos adotados. Ajustar os coeficientes das pegadas ambientais de acordo com forma como os alimentos são consumidos reduziu as possibilidades de erros e inconsistências^24^. Além disso, o cálculo dos impactos ambientais das dietas por quantidades fixas de calorias propiciou a comparação adequada entre os padrões dietéticos por controlar os efeitos das diferenças na quantidade de alimentos consumida.

## CONCLUSÃO

A pegada de carbono da dieta brasileira, estimada por nosso estudo em 4.489g CO2e/ pessoa/dia, excede em cerca de 30% a pegada da dieta humana que poderia simultaneamente atender os requisitos nutricionais de uma dieta saudável e a meta global de contenção do aumento da temperatura média do planeta. O padrão de associação entre variáveis sociodemográficas e as pegadas de carbono da dieta descrito neste estudo pode auxiliar na identificação de alvos prioritários para ações públicas que visem a reduzir os impactos ambientais do consumo alimentar no Brasil.
